# Y chromosome evidence of earliest modern human settlement in East Asia and multiple origins of Tibetan and Japanese populations

**DOI:** 10.1186/1741-7007-6-45

**Published:** 2008-10-29

**Authors:** Hong Shi, Hua Zhong, Yi Peng, Yong-Li Dong, Xue-Bin Qi, Feng Zhang, Lu-Fang Liu, Si-Jie Tan, Runlin Z Ma, Chun-Jie Xiao, R Spencer Wells, Li Jin, Bing Su

**Affiliations:** 1 State Key Laboratory of Genetic Resources and Evolution, Kunming Institute of Zoology and Kunming Primate Research Centre, Chinese Academy of Sciences, Kunming, PR China; 2 Institute of Genetics and Developmental Biology, Chinese Academy of Sciences, Beijing, PR China; 3 Human Genetics Centre, Yunnan University, Kunming, PR China; 4 State Key Laboratory of Genetic Engineering and Center for Anthropological Studies, School of Life Sciences, Fudan University, Shanghai, PR China; 5 Huaihua Medical College, Huaihua, Hunan, PR China; 6 The Genographic Project, National Geographic Society, Washington, USA

## Abstract

**Background:**

The phylogeography of the Y chromosome in Asia previously suggested that modern humans of African origin initially settled in mainland southern East Asia, and about 25,000–30,000 years ago, migrated northward, spreading throughout East Asia. However, the fragmented distribution of one East Asian specific Y chromosome lineage (D-M174), which is found at high frequencies only in Tibet, Japan and the Andaman Islands, is inconsistent with this scenario.

**Results:**

In this study, we collected more than 5,000 male samples from 73 East Asian populations and reconstructed the phylogeography of the D-M174 lineage. Our results suggest that D-M174 represents an extremely ancient lineage of modern humans in East Asia, and a deep divergence was observed between northern and southern populations.

**Conclusion:**

We proposed that D-M174 has a southern origin and its northward expansion occurred about 60,000 years ago, predating the northward migration of other major East Asian lineages. The Neolithic expansion of Han culture and the last glacial maximum are likely the key factors leading to the current relic distribution of D-M174 in East Asia. The Tibetan and Japanese populations are the admixture of two ancient populations represented by two major East Asian specific Y chromosome lineages, the O and D haplogroups.

## Introduction

**Figure 1:**
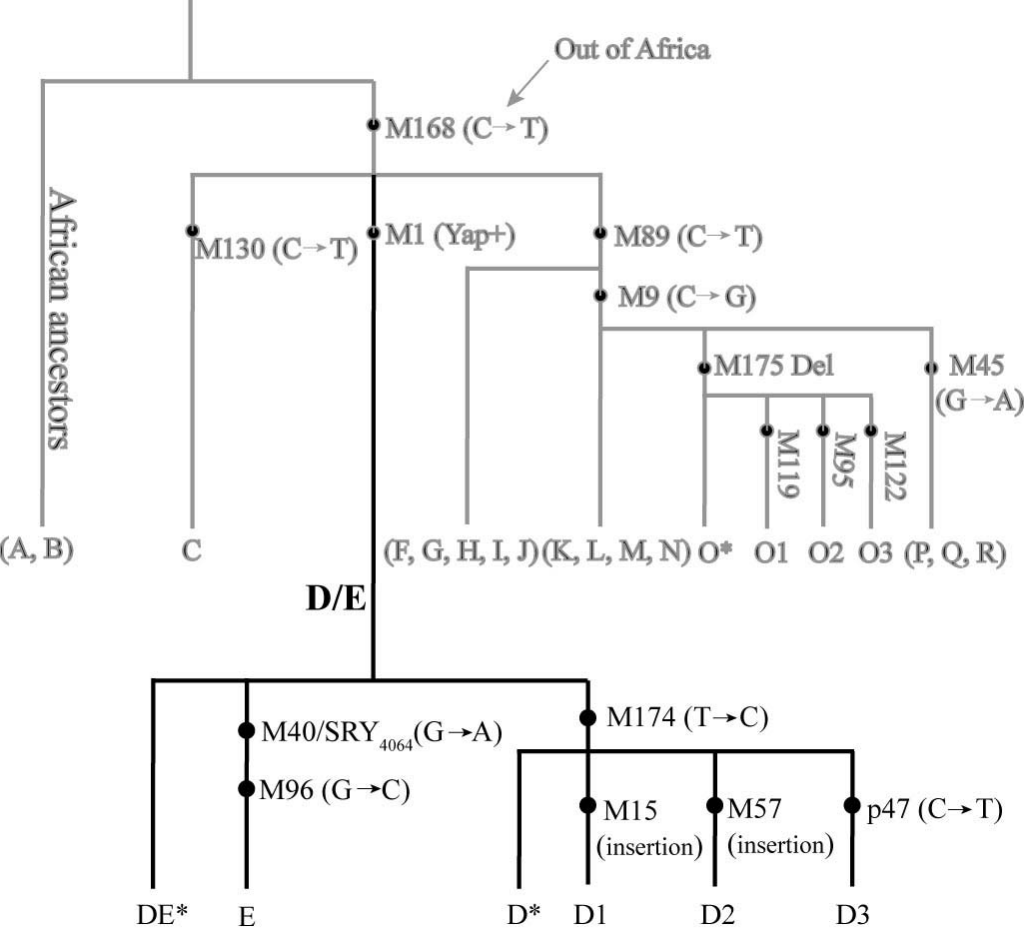
The phylogenetic relationship of the D-M174 lineages and the Y chromosome biallelic markers defining these lineages.

The Y chromosome *Alu *polymorphism (YAP, also called M1) defines the deep-rooted haplogroup D/E of the global Y-chromosome phylogeny [1]. This D/E haplogroup is further branched into three sub-haplogroups DE*, D and E (Figure 1). The distribution of the D/E haplogroup is highly regional, and the three subgroups are geographically restricted to certain areas, therefore informative in tracing human prehistory (Table 1). The sub-haplogroup DE*, presumably the most ancient lineage of the D/E haplogroup was only found in Africans from Nigeria [2], supporting the "Out of Africa" hypothesis about modern human origin. The sub-haplogroup E (E-M40), defined by M40/SRY_4064 _and M96, was also suggested originated in Africa [3-6], and later dispersed to Middle East and Europe about 20,000 years ago [3,4]. Interestingly, the sub-haplogroup D defined by M174 (D-M174) is East Asian specific with abundant appearance in Tibetan and Japanese (30–40%), but rare in most of other East Asian populations and populations from regions bordering East Asia (Central Asia, North Asia and Middle East) (usually less than 5%) [5-7]. Under D-M174, Japanese belongs to a separate sub-lineage defined by several mutations (*e.g. *M55, M57 and M64 *etc.*), which is different from those in Tibetans implicating relatively deep divergence between them [1]. The fragmented distribution of D-M174 in East Asia seems not consistent with the pattern of other East Asian specific lineages, *i.e. *O3-M122, O1-M119 and O2-M95 under haplogroup O [8,9].

**Table 1:** The reported frequency distribution of Yap+(M1) in world populations.

Population	Samples	M96	M174	M15	M57	Population	Samples	M96	M174	M15	M57
Africans	1,848	72.08				Tibetans	472		29.45	11.86	
Europeans	2,398	11.68				Hmong-Mein	199			4.02	
Native Americans	371	1.62				Tibeto-Buman	868		5.88	3.57	
Russians	301	2.66				Daic	68			1.47	
Mid-east	1,970	10.05	0.10			Northern Han	521		0.96	0.38	
Central Asia	1,325	4.53	0.83			Southern Han	1,373		0.87	0.29	
Indians	996	0.80				Northwest Han	201		4.48		
Sri Lankans	83					Taiwanese	152		0.66		
Northeast Indian	232		0.86			Philipines	77				
Australasians	246					Thais	40		10.00		
North Asians	908		0.33			Indonesians	100				
Nepal	98		4.08			Malaysian	45			2.22	
Japanese	516				35.08	Southeast Asians	387		0.78	1.03	
Koreans	315		3.81			Andamanese	48		56.25		
Uygurs	68		4.41			Melanesia	113				
Mongolians	303		1.98	0.66		Micronesia	73		1.37		
Manchurian	70					Polynesia	40				
Hui	74		5.41	2.70		Total	16,859				

Note: the frequency data of YAP+ was from published studies [4-7,9-11,13,14,16-18,23,27-29,43-50].

Besides Tibetans and Japanese, D-M174 is also prevalent in several southern ethnic populations in East Asia, including the Tibeto-Burman speaking populations from Yunnan province of southwestern China (14.0–72.3%), one Hmong-Mien population from Guangxi of southern China (30%) and one Daic population from Thailand (10%), which could be explained by fairly recent population admixture [9-11]. However, a recent study reported a high frequency of D-M174 in Andamanese (56.25%), people who live in the remote islands in the Indian Ocean and considered one of the earliest modern human settlers of African origin in Southeast Asia [12]. Another study by Underhill *et al. *suggested that the D-M174 lineage likely reached East Asia about 50,000 years ago [5]. This implies that the YAP lineage in East Asia could be indeed very ancient.

Our previous studies showed that the dominant and East Asian specific Y haplogroup O-M175 (44.3% on average) reflected possibly the earliest modern human expansion in East Asia [8,9,13]. Unlike the prevalence of O-M175 in most of the East Asian populations, populations with relatively high frequencies of D-M174 are mostly located at the peripheral regions of mainland East Asia with fragmented distribution [7,9-11,13-18], implying two possible explanations of human prehistory. Firstly, like O-M175, D-M174 may also be just one of the lineages going northward during the suggested Paleolithic migration of modern humans in East Asia [8,9]. Subsequently, due to population substructure (the last glacial is likely a key factor) and recent expansion of Han culture [18], the distribution of D-M174 was fragmented into the current geographic pattern. The other possible scenario would suggest an independent earlier migration different from the one we proposed previously [8,9]. To address this question, we conducted a systematic sampling and genetic analysis of more than 5,000 male individuals from 73 East Asian and Southeast Asian populations. Based on the Y chromosome SNP and STR data and the estimated ages of the major D-M174 lineages, we proposed that there was an independent Paleolithic northward migration of modern humans in East Asia, predating the previously suggested northward population movement [8,9,13,19-21].

## Methods

### Samples

**Figure 2:**
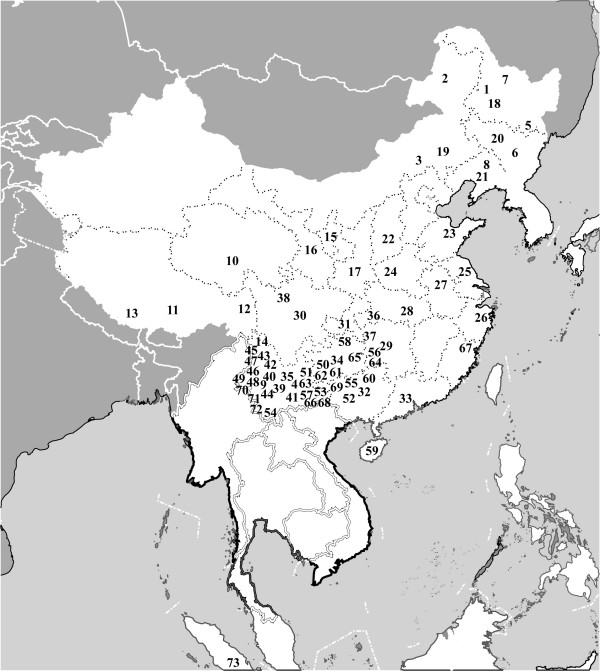
The geographic locations of the 73 populations (numbers in black) sampled in this study.

In this study, a total of 5,134 unrelated male samples were collected from 73 populations, covering the major geographic regions in East Asia and Southeast Asia (Table 2 and Figure 2). As shown in Figure 2, most populations were sampled from southern and southwestern China where about 80% Chinese ethnic populations live with inhabited histories longer than 3,000 years [22]. Samples from previous studies were also included, including 91 YAP+ samples (16 Japanese, 54 Tibetans, 3 Koreans, 1 Guam, 1 Cambodian, 4 Thais and 12 samples of China) from Su *et al. *[7,9,17] and 116 YAP+ samples (35 Africans, 13 Caucasians, 9 Indians, 26 Middle East and 33 Central Asians) from Wells *et al. *[23](Table 2).

**Table 2:** The sub-haplogroup/haplotype distribution of D-M174 in eastern Asia.

	**Samples**	**Language Family**	**Sample Sites**	**Size**	**Frequency of the haplotypes (%)**						
					
					**Yap+**	DE*	D*-M174	**D1-M15**	**D2-M57**	**D3-p47**	**E-M40**
North East Asians	Japanese	Altai	*	49	32.60				32.60		
	Korean	Altai	*	45	6.60		4.40	2.20			
	Daur	Altai	1	4							
	Ewenki	Altai	2	31							
	Mongol	Altai	3, 4	87							
	Korea	Altai	5, 6	46							
	Manchu	Altai	7, 8, 9	125	1.60		0.80	0.80			
	Tibetan	Tibeto-Burman	10, 11, 12, 13, 14	594	47.47	0.34	2.36	10.10		34.68	
	Tibetan	Tibeto-Burman	*	128	42.10		7.00	23.40		11.70	
	Mongol	Altai	*	24	4.10					4.10	
	Hui	Altai	15	24	4.17		4.17				
	Northwestern Han	Han	16, 17	614	6.51			3.26		2.61	0.65
	Northern Han	Han	18, 19, 20, 21, 22, 23, 24	454	1.54			1.54			
South East Asians	Southern Han	Han	25, 26, 27, 28, 29, 30, 31, 32, 33, 34, 35	768	1.43			1.43			
	Southern Han	Han	*	283	1.40		1.40				
	Tujia	Tibeto-Burman	36, 37	116	2.59			2.59			
	Qiang	Tibeto-Burman	38	27	29.63			29.63			
	Yi	Tibeto-Burman	39	54	3.70			3.70			
	Bai	Tibeto-Burman	40	199	2.51		1.51	1.01			
	Hani	Tibeto-Burman	41	42							
	Naxi	Tibeto-Burman	42	87	32.18			2.30		29.89	
	Pumi	Tibeto-Burman	43	47	72.34			2.13		70.21	
	Lahu	Tibeto-Burman	44	78							
	Dulong	Tibeto-Burman	45	30							
	Lisu	Tibeto-Burman	46	50							
	Nu	Tibeto-Burman	47	52	1.92		1.92				
	Achang	Tibeto-Burman	48	40							
	Jingpo	Tibeto-Burman	49	50	2.00			2.00			
	Buyi	Daic	50, 51	123	2.44			2.44			
	Chuang	Daic	52, 53	116	10.34		2.59	7.76			
	Chuang	Daic	*	28	3.50			3.50			
	Dai	Daic	54	176	4.55		1.70	2.27		0.57	
	Dong	Daic	55, 56, 57	147	3.40			3.40			
	Gelo	Daic	58	14							
	Li	Daic	59	55	5.45			5.45			
	Mulam	Daic	60	78	6.41			6.41			
	Maonan	Daic	61	15							
	Shui	Daic	62, 63	95	5.26			5.26			
	Miao	Hmong-Mien	64, 65, 66	212	4.72			4.72			
	She	Hmong-Mien	67	12							
	Yao	Hmong-Mien	68, 69	284	11.27			11.27			
	Yao	Hmong-Mien	*	20	25.00			25.00			
	De'ang	Austro-Asiatic	70	45							
	Wa	Austro-Asiatic	71	31							
	Bulang	Austro-Asiatic	72	29							
	Guam	Austronesian	*	6	16.60		16.60				
	Thai	Daic	*	40	10.00		10.00				
	Cambodian	Austronesian	*	26	3.80			3.80			
	Indonesian	Austronesian	73	83							
	Total			5783	10.30						
Control population	Central Asian		*	984	3.30		0.10	0.50		0.30	2.40
	Caucasus		*	147	8.80			0.70			8.10
	Middle East		*	102	25.40						25.40
	Indian		*	261	3.40						3.40
	African		*	44	79.50						79.50

Note: * the Yap+ samples from Su et al. 1999, 2000a, 2000b and Wells et al. 2001. The numbers of sample sites can be found in Figure 2.

### Y Chromosome Markers and Genotyping

Initially all the samples were genotyped for three Y chromosome specific biallelic markers, including M1, M40 and M174. The samples belonging to sub-haplogroup D (D-M174) were subject to further typing of M15, M57 and p47 in order to designate the sub-clades. The sub-haplogroups were named by the characteristic mutations according to the Y-Chromosome-Consortium (2002). The PCR electrophoresis, PCR-RFLP and sequencing were used for genotyping [7]. The phylogenetic relationship of the Y biallelic markers is shown in Figure 1.

We typed the YAP locus in the 5,134 samples, of which 512 YAP+ were detected (9.97%), plus the previously published 207 Yap+ samples, and a total 719 Yap+ samples were then typed for the five biallelic markers (M174, M40, M15, M57 and p47) and eight STRs (DYS19/394, DYS388, DYS 389I, DYS389II, DYS 390, DYS 391, DYS 392 and DYS 393). Among the 719 YAP+, a total of 697 samples generated complete sets of allele counts for all the SNPs and STRs (data in the Additional files).

### Data Analysis

For data analysis, we included the YAP+ samples from the published data on 90 Japanese and 44 Tibetans from Hammer *et al. *(2006), 19 Andamanese from Thangaraj *et al. *(2003) and 6 Nigerians from Weale *et al. *(2003). The divergence times between the sub-clades of D-M174 were estimated using the STR data following the SNP-STR coalescence method [4,24,25]. The average mutation rate of the Y-STRs tested is 0.00069 [26]. The network of the Y-STR haplotypes were constructed for each D-M174 sub-haplogroup using NETWORK4.2.0.1 , and then superimposed onto the established phylogeny of the D-M174 lineage (Figure 1). The average gene diversity of the populations was calculated with the use of the allele frequencies of the eight tested STR loci (Arlequin 3.0, ).

## Results

Table 1 lists the reported YAP+ frequencies in worldwide populations (refer to table note for references). Africans have the highest frequency of YAP+, and all of them belong to the sub-haplogroup E-M40. In contrast, D-M174 is in general East Asian specific with sporadic occurrence in adjacent regions, *i.e. *Central Asia, Middle East and Northeast India. The average frequency of D-M174 in East Asians is 9.60% with high frequencies in Tibet (41.31%), Japan (35.08%) and Andaman Island (56.25%), but rare in other East Asian populations (< 5%). After genotyping the Y chromosome biallelic markers (M174, M40, M15, M57 and p47), the 719 Yap+ samples were assigned to 6 sub-haplogroups, *i.e. *DE*, E-M40, D*-M174, D1-M15, D2-M57 and D3-p47 (Figure 1). Further typing for eight STR loci of the 719 YAP+ generated the complete STR data sets for 697 samples. As shown in Table 2, consistent with previous reports [7,9-11,13,16], the prevalence of D-M174 is mostly in western and southern China and Japan.

The distribution patterns of the four D-M174 sub-lineages (sub-haplogroups) (Figure 1) are different from each other. D1-M15 is widely distributed across East Asia including most of the Tibeto-Burman and Daic speaking populations (Table 2). D*-M174 and D3-p47 are mainly distributed in Tibeto-Burman populations with sporadic occurrence in the Daic populations. In surprise, we observed two DE* in the Tibetan samples, which was previously only observed in Africa (Nigerians), but not in other world populations. In contrast, D2-M57 only occurred in Japanese, an implication of the early divergence of this lineage from other D-M174 sub-haplogroups (Table 2). We identified four E-M40 individuals in the northwestern Han populations, a reflection of recent gene flow from Central Asia [23].

**Figure 3:**
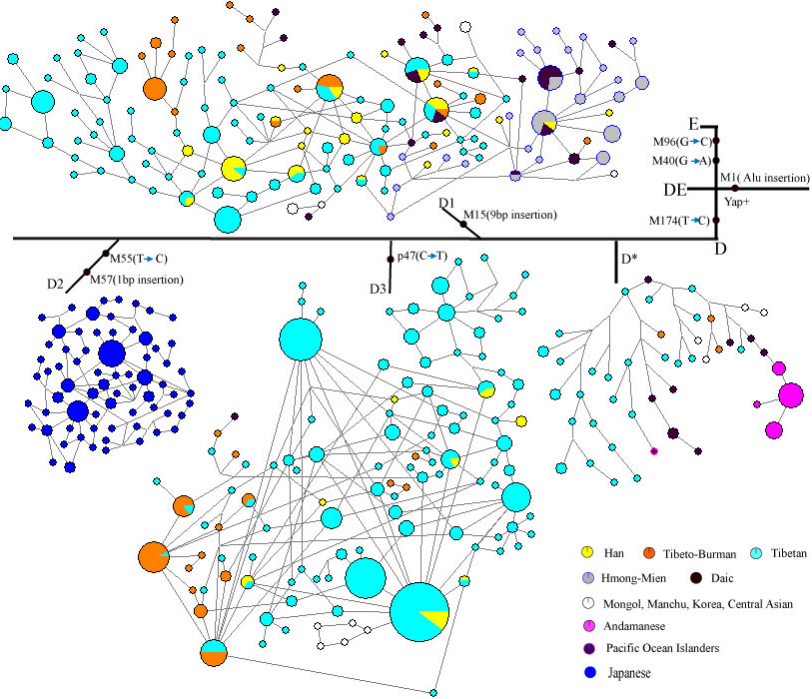
**The Y-STR network of the D-M174 lineages.** The Tibeto-Burman populations do not include Tibetans. The southern ethnic populations (Daic and Hmong-Mien) form a relatively separate cluster from Tibetan and Tibeto-Burman populations in the D1-M15 sub-haplogroup.

To reveal the detailed structure of the D-M174 lineage in East Asian populations, we conducted the network analysis combining the SNP and STR haplotype data (Figure 3). D*-M174 has a deep structure with no loops in the network. The D*-M174 lineage contains distinct STR haplotypes of Tibeto-Burman (mostly Tibetan), Daic and Andamanese respectively, and no haplotype sharing (i.e. shared by individuals from different geographic/linguistic populations) was observed, implying that D*-M174 is an ancient lineage. As the most common lineage, the network of D1-M15 is also deep-structured and a clear south vs. north divergence can be inferred though sporadic haplotype sharing exists. In contrast, D2-M57 is restricted to Japan and D3-p47 is prevalent in Tibet and its adjacent regions with sporadic appearance in Central Asian and Daic populations. The short-distanced and star-like network structure of these two sub-haplogroups indicates a long-term local existence and population expansion of the D-M174 lineage in these two geographically far away regions. The non-Tibetan Tibeto-Burman populations, *e.g. *Naxi, Pumi and Qiang only have a subset of the Tibetan haplotypes, again indicating a recent gene flow from Tibet as recorded in the literature [7,22].

We next sought to estimate the age of the D-M174 sub-lineages. The coalescence analysis shows that the age of D*-M174, D3-p47 and D1-M15 are the oldest (66,392 ± 1,466, 52,103 ± 1,327 and 51,640 ± 2,563 years). The age of the Japanese specific lineage D2-M57 is the youngest (37,678 ± 2,216 years) (Table 3). Notably, these estimated ages are much older than that of O3-M122, the other East Asian specific haplogroup we reported before (25,000–30,000 years) [8].

**Table 3:** Estimated divergence times of the D-174 sub-lineages

	**DIVERGENCE TIME (YEARS AGO)**		
	
**D_M174 SUBLINEAGE**	***Upper Limit***	***Lower Limit***	***Mean *± *SE***
D*_M174	71,365	61,418	66,392 ± 1,466
D1_M15	67,565	35,715	51,640 ± 2,563
D3_p47	68,028	36,179	52,103 ± 1,327
D2_M57	53,603	21,753	37,678 ± 2,216

Note: The divergence times were estimated in accordance with the method of TD [25,26]. The upper limit was determined with the assumption that *V*_0 _= 0 [24]. The lower limit was determined with the assumption that *V*_0 _= *V*_*a*_, where *V*_*a *_is the value of within-population variance in the ancestral populations.

## Discussion

The biased distribution of D-M174 bears the different possible inferences of the human population prehistory in view of the origin and migratory pattern in East Asia. The hypothesis of northern origin of D-M174 is not supported by our data because D-M174 is rare in Central Asian populations (Table 1) and the few Central Asian D-M174s are all located at the peripheral positions of the Y-STR network (Figure 3). Our data also disapproves the notion of Indian origin since no D-M174 was detected in the 996 individuals across India. The sporadic occurrence of D-M174 in northeastern Indians (two D-M174s in 232 individuals tested) is because those populations are in fact Sino-Tibetan speaking populations (Table 1). The lack of gene flow between Tibet and India is likely due to the efficient geographic separation by the Himalayas [27].

On the other hand, the aborigines living on Andaman Island are genetically isolated in view of their Y chromosome haplotypes. Though phenotypically different from other Southeast Asian populations, the Andaman Islanders posses most of the major East Asian specific Y chromosome lineages including D-M174, O3-M122 and O2-M95, a strong indication of a relic Paleolithic population [28]. Also, the Daic and Hmong-Mien speaking populations are ancient southern populations in view of linguistic and archaeological evidences [22]. The network analysis indicates a clear divergence of D1-M15 between northern (Tibeto-Burman) and southern (Daic and Hmong) populations (Figure 3). Hence, the alternative explanation of northern origin of D-M174 is unlikely considering the absence of YAP+ in North Asia [29] and the sporadic appearance of D-M174 in Central Asia [23]. Consequently, the southern origin of D-M174 can be established, which is consistent with the proposed initial settlement of modern humans in mainland Southeast Asia and the migration pattern of other Y chromosome lineages [8,9,13].

There were studies arguing against the southern origin of East Asian, in which higher gene diversity was observed in northern populations compared with southern populations [14,30]. As we discussed in our previous report, the data from Karafet *et al*. [14] suggested a false impression of the high diversity of northern populations without taking the recent admixture from Central Asia into consideration [8]. The study of Xue *et al. *[30] has the similar drawback though both Y-SNP and Y-STR data were used. In Xue *et al *(2006), the high gene diversity was claimed for Mongolian, Uygure and Manchurian, and all of them have recorded recent extensive admixture with Central Asian and Han Chinese populations [22]. In addition, the southern populations studied in Xue *et al. *(2006) were limited and the within population bottleneck effect caused by long-time geographic isolation might have a great impact on gene diversity estimation. When plenty southern populations were studied, we observed a higher diversity in those populations compared with the northern populations [8,9].

The gene diversity based on the STR data in the southern populations is comparable with those in the northern populations. Tibetan has the highest diversity (0.525 ± 0.294), followed by Daic (0.484 ± 0.272), Japanese (0.419 ± 0.239) and Hmong Mien (0.347 ± 0.206). Gene diversity of other East Asian populations was not calculated due to small sample size. The higher diversity in Tibetan is largely due to the much larger effective population size of D-M174 in Tibetan compared with other populations. Tibetan and Japanese lived in two geographically far away regions and their D-M174 lineages belong to two different sub-haplogroups. These two sub-haplogroups all have a short-distanced and star-like network structure, which indicates a long-term local existence and recent population expansion (Figure 3). It should be noted that the estimation of gene diversity is subject to potential bias, e.g. the age difference of the D-M174 sub-lineages. The finding of two DE* in Tibet, which was only observed in Africa, supports the antiquity of D-M174 and suggests that the D-M174 lineage is among the earliest modern human settlers in East Asia. Additionally, the biased distribution of D-M174 and its ancient coalescent time suggests an independent Paleolithic migration of modern humans in East Asia.

Our previous data on the O3-M122 lineage suggested a prehistorically northward migration (about 25,000–30,000 years ago) of modern humans in East Asia, which explains the current phylogeography of the major East Asian specific Y chromosome lineages (O3-M122, O2-M95 and O1-M119) [8,9,13]. However, the data on D-M174 cannot be explained by the hypothesized migration pattern. Firstly, D-M174 is rare in the central part of eastern Asia, especially in Han Chinese populations. Though this could be explained by genetic drift, assuming D-M174 moving along with O3-M122 during the proposed northward migration, the prevalence of D-M174 in Tibet and Japan requires recurrent mutations or independent random enrichment of D-M174, which is unlikely. An independent earlier northward migration is more reasonable in explaining the current distribution of D-M174 in East Asia. We speculate that due to the later northward migration of O3-M122 and the Neolithic expansion of Han Chinese, the trace of the D-M174 migration in the heartland of East Asia was wiped out by the later and likely much larger migration of O3-M122. The current peripheral distribution pattern of D-M174 in East Asia is consistent with the proposed notion. Also, the age estimation supports that the northward migration of D-M174 may predate the migration of O3-M122.

The East African megadroughts (about 135–75 thousand years ago) during the early late-Pleistocene was suggested compelling modern humans out of Africa [31]. And the early modern human could occupy coastal areas and exploited the near-shore marine food resource by that time [32]. Then, modern humans was suggested expanding along the tropical coast, and the earliest modern human fossil found out of Africa was about 100,000 years ago [33]. The period of 80,000–10,000 years ago during the last glacial might have a huge impact on modern human migration, and the sea level had fallen 50–200 meters below present [34], which resulted in larger dry lands and possibility for human migration between currently separated lands by ocean, *e.g. *between Japan and the mainland.

Human fossil records and previous genetic data suggested that the earliest modern human settlement in East Asia likely occurred less than 60,000 years ago [8,9,13,21,35]. For example, the oldest Australian fossil (Lake Mungo 3) was dated about 45,000 ± 3,000 – 62,000 ± 6,000 years ago [36,37], and the mitochondrial DNA and Y chromosome analysis of current Australian ethnic populations suggested the colonization of Australia about 50,000–70,000 years ago [38]. Our age estimation of the D-M174 lineages is consistent with this notion though there might be independent migrations of modern humans into East Asia and into Australia [38].

The estimated ages of the D-M174 lineages are older than those previously reported based on both Y chromosome and mtDNA variations in East Asia [8,9,21]. To see whether it is over-estimated, using the same method, we calculated the divergence time between DE* and E-M40. The estimated age is 27,176 years, which is much younger than the D-M174 lineage, but consistent with the previous estimation (27,800–37,000 years ago) [3]. Hence, the antiquity of D-M174 likely reflects the true prehistory of human populations in East Asia. The age estimation model developed by Zhivotovsky (2001) is not sensitive to effective population size and recent population expansion though the effect of population substructure cannot be totally ruled out. The antiquity of D-M174 was also supported by a previous study in which the origin of D-M174 was estimated more than 50,000 years ago [5].

The divergence time of haplogroup D is about 60,000 years ago, considering the wide though fragmented geographic distribution of D-M174, the proposed Paleolithic migration would be the first northward population movement of modern humans after their initial settlement in southern East Asia. As the last glacial occurred during 80,000–10,000 years ago, the northward migration of D-M174 is consistent with the proposed notion that modern humans might exploit the food of "Mammoth Steppe" [39]. Besides the later population expansion, the cold weather during the last glacial may also contribute to the current fragmented distribution of D-M174. Interestingly, a recent archaeological finding supported that modern humans explored the Tibetan plateau about 30,000–40,000 years ago, which is much earlier than previously suggested [40], but consistent with our hypothesis. The after-glacial sea level rise eventually led to the separation between Japan and the main continent, which explains the relic distribution of D-M174 in current Japanese populations. The archaeological data suggested that the initial colonization of modern humans in Japan occurred about 30,000 years ago [41,42], consistent with our age estimation of D2-M57 (37,678 ± 2,216 years ago). Taken together, the current Tibetan and Japanese populations are probably the admixture of two ancient populations represented by D-M174 and O3-M122 respectively [7,10,16].

## Conclusion

In summary, we demonstrated an ancient Paleolithic population migration in East Asia, predating the previously suggested northward population movement. The current fragmented distribution of D-M174 is likely due to the combination of later Neolithic population expansion and the last glacial.

## Authors' contributions

BS and LJ designed research. BS and HS analyzed data and wrote the paper. HS, HZ, YP, Y-LD and X-BQ performed research. FZ, LF-L, S-JT, R-LM, C-JX and SW provided some research samples.
